# Modeling Interactions between Speech Production and Perception: Speech Error Detection at Semantic and Phonological Levels and the Inner Speech Loop

**DOI:** 10.3389/fncom.2016.00051

**Published:** 2016-05-31

**Authors:** Bernd J. Kröger, Eric Crawford, Trevor Bekolay, Chris Eliasmith

**Affiliations:** ^1^Neurophonetics Group, Department of Phoniatrics, Pedaudiology, and Communication Disorders, Medical School, RWTH Aachen UniversityAachen, Germany; ^2^Reasoning and Learning Lab, School of Computer Science, McGill UniversityMontreal, QC, Canada; ^3^Centre for Theoretical Neuroscience, University of WaterlooWaterloo, ON, Canada

**Keywords:** neurocomputational model, speech production and perception, inner speech, spiking neural networks, speech errors

## Abstract

Production and comprehension of speech are closely interwoven. For example, the ability to detect an error in one's own speech, halt speech production, and finally correct the error can be explained by assuming an inner speech loop which continuously compares the word representations induced by production to those induced by perception at various cognitive levels (e.g., conceptual, word, or phonological levels). Because spontaneous speech errors are relatively rare, a picture naming and halt paradigm can be used to evoke them. In this paradigm, picture presentation (target word initiation) is followed by an auditory stop signal (distractor word) for halting speech production. The current study seeks to understand the neural mechanisms governing self-detection of speech errors by developing a biologically inspired neural model of the inner speech loop. The neural model is based on the Neural Engineering Framework (NEF) and consists of a network of about 500,000 spiking neurons. In the first experiment we induce simulated speech errors semantically and phonologically. In the second experiment, we simulate a picture naming and halt task. Target-distractor word pairs were balanced with respect to variation of phonological and semantic similarity. The results of the first experiment show that speech errors are successfully detected by a monitoring component in the inner speech loop. The results of the second experiment show that the model correctly reproduces human behavioral data on the picture naming and halt task. In particular, the halting rate in the production of target words was lower for phonologically similar words than for semantically similar or fully dissimilar distractor words. We thus conclude that the neural architecture proposed here to model the inner speech loop reflects important interactions in production and perception at phonological and semantic levels.

## Introduction

Speech production is a hierarchical process starting with the activation of an idea, which is intended to be communicated, proceeds with the activation of words, then with modification and sequencing of words with respect to grammatical and syntactic rules, and ends with the activation of a sequence of motor actions that realize the intended utterance (Dell and Reich, 1981; Dell et al., 1997; Levelt et al., 1999; Levelt and Indefrey, 2004; Riecker et al., 2005). Despite the complexity and depth of the speech production hierarchy, the production process runs nearly error free. Speech errors occur relatively seldom and typically need to be evoked in experiments if we want to study them (Levelt, 1983; Nooteboom and Quené, 2015). This robustness supports the assumption that speech production benefits from a robust neural mechanism for activating and processing already learned and stored cognitive and sensorimotor speech units (e.g., syllables, words, short phrases). Additionally, this robustness supports the assumption that speech production may be monitored at different levels in order to detect and repair occurring errors (Postma et al., 1990; Postma, 2000; Hartsuiker and Kolk, 2001; Schwartz et al., 2016).

Restricting our attention to single word production (such as in a picture naming task), speech production starts with the activation of semantic concepts (e.g., “has wheels,” “can move,” “can transport persons,”), retrieves an associated word (e.g., “car”) and its phonological form (/kar/) from the mental lexicon (see e.g., Dell and Reich, 1981; Levelt et al., 1999), activates the relevant motor plan, which can be thought of as a collection of intended speech movements (such as: form tongue, lower jaw and lips for /k/, then for /ar/; in parallel open glottis for production of the unvoiced speech sound /k/ and then for the voiced sound /ar/) and then executes these speech movements or actions in order to articulate the intended word and to generate the appropriate acoustic signal (see e.g., Kröger and Cao, 2015). This production process mainly consists of two stages, one cognitive and one sensorimotor. The *cognitive stage* consists of concept activation, word selection and the subsequent activation of the related phonological representation (Dell et al., 1997; Levelt et al., 1999), while the *sensorimotor stage* consists of motor plan activation (also called motor planning) and execution (Riecker et al., 2005; Kröger and Cao, 2015).

Both the cognitive and sensorimotor stages of speech production mainly involve retrieving and activating units or chunks already stored in repositories of cognitive knowledge and sensorimotor skill, respectively. This knowledge and these skills were learned during speech and language acquisition. The cognitive knowledge repository that plays a central role in word production is called the *mental lexicon*. Here, a neural word node (also called lemma node) is associated both with a semantic or conceptual representation of the word and a lexical or phonological representation of the word (Levelt et al., 1999). The sensorimotor skill repository is called the *mental syllabary*. Within this repository, phonological forms of syllables or (short) words are associated with motor plans, as well as with auditory and somatosensory mental images of the already acquired syllable or word (Kröger and Cao, 2015). Monitoring at the sensorimotor level is mainly a matter of comparing learned auditory and somatosensory images with the sounds generated during speech articulation, which are fed back through the auditory system (auditory self-perception). This monitoring process is slow, because it includes both motor execution and auditory perception (Postma, 2000). A faster monitoring loop called the *inner speech loop* compares word representations activated at the cognitive level of the production hierarchy to those activated by a level of the perception hierarchy, here better labeled as comprehension. This monitoring mainly consists of comparing the intended conceptual and phonological representations with the instantaneously-activated conceptual and phonological representations evoked during speech production, and leads to *inner self-perception* (Hartsuiker and Kolk, 2001). Inner self-perception assumes the existence of inner speech (also called covert speech), while auditory self-perception or outer self-perception requires the production of audible speech, also called overt speech (Oppenheim and Dell, 2008).

It can be assumed that speech monitoring, i.e., the comparison of intended and produced speech, can be realized by linking production and perception outcomes at different levels or stages (e.g., concept, phonological form, or motor plan levels). While the slower outer speech loop includes all stages (from conceptualization to articulation and back), the inner speech loop only includes conceptualization until retrieval of the phonological form and vice versa. This inner loop theory of speech monitoring has been successful in explaining the fact that speech errors are often repaired so quickly that the involvement of the (slow) auditory feedback loop (outer speech loop) can be ruled out (Postma, 2000; Hartsuiker and Kolk, 2001).

Because it is not trivial to evoke speech errors, in this study a picture naming and halt paradigm is used (Slevc and Ferreira, 2006). In this paradigm, utterance of a target word is elicited by normal picture naming, where picture-to-word associations are pre-learned in an initial familiarization procedure (Slevc and Ferreira, 2006, p. 520). About 400 ms later, an acoustically presented halt signal (distractor word) is presented and subjects are required to stop production of the target word if the distractor word is different from the target word. One significant finding of the picture naming and halt study performed by Slevc and Ferreira (2006) was that the mean stopping rate depends on the phonological similarity between the target and distractor word, but not on the semantic similarity. Semantically similar distractor words were found to have the same stopping accuracy as distractor words dissimilar from the target word (Slevc and Ferreira, 2006, p. 521). It should be noted that this paradigm does not directly relate to speech error detection, but is a method for investigating the mechanisms governing speech monitoring. A second main result of the experiment described by Slevc and Ferreira (2006) is that the speech monitor is capable of detecting differences more easily in the case of distractor words which are completely dissimilar to the target word as well as to distractor words which are semantically similar, while phonologically similar distractor words are not detected as easily. The halting rate for phonologically similar words was found to be the lowest.

The main goal of this study is to develop a neural architecture for speech production and perception (and comprehension), which, on the one hand, enables fast, effortless and error-free realization of word production, and, on the other hand, allows for the simulation of speech errors and realistic and effective speech monitoring. Thus, the neural architecture of our model consists of speech production, perception and monitoring components and, furthermore, should be capable of detecting and correcting speech errors. A further major goal of this study is to underline the tight connection between speech production and perception (e.g., Pickering and Garrod, 2013).

The neural model of speech processing developed here uses the principles of the Neural Engineering Framework (NEF, Eliasmith and Anderson, 2003; Eliasmith, 2013). We used this framework because it allows for the development of neurobiologically plausible large-scale models of both cognitive and sensorimotor components, and because it has already been shown to be capable of producing models that match human performance on a number of non-speech behavioral tasks (Eliasmith et al., 2012). Three basic principles characterize the NEF: representation, transformation, and dynamics (Eliasmith and Anderson, 2003). (i) Representation means that the NEF allows to code external (sensory or motor) signals as *neural states* and to decode neural states as non-neural (external physical) signals. These neural states are represented within the NEF as *neural activation patterns* or *spike patterns* of specific neuron ensembles. Thus, a *neuron ensemble* is the basic unit within the NEF for representing neural states. Each neural ensemble consists of a specific number of individual neurons; in this study *leaky integrate-and-fire (LIF) neurons* are used. (ii) Transformation means that neuron ensemble A can be connected to a downstream neuron ensemble B by establishing a neural connection from each neuron within ensemble A to each neuron within ensemble B. These *neural connections* not only allow *communication of a neural state* from A to B, but can also be constructed to transform the neural state represented by A into a neural state in B that is a function of the neural state represented by A. (iii) Dynamics means that a neural state of a neuron ensemble changes with respect to input over time. Input can be provided by other neuron ensembles, or by the same neuron ensemble through *recurrent connections*. Here, the each neuron within the neuron ensemble is connected to the other neurons in the neuron ensemble. This allows for the neural state to maintained in the absence of input, implemented a type of *neural memory*.

A model of speech production, speech perception, speech monitoring, and speech error detection and repair is complex and must include cortical as well as subcortical components. For building complex models using the NEF it is advantageous to use the *Semantic Pointer Architecture* (SPA, Eliasmith, 2013; Stewart and Eliasmith, 2014; Gosmann and Eliasmith, 2016). The SPA is based on the NEF and allows for more complex neural representations and transformations than would otherwise be possible. In the SPA, complex neural states are called *semantic pointers* (SPs). Semantic pointers (e.g., “A,” “B,” and “C”) are capable of representing different cognitive states, for example semantic concepts (e.g., A = ‘Is_Blue’, B = ‘Has_Four_Legs’, C = ‘Apple’, Eliasmith, 2013; Blouw et al., 2015), or orthographic and phonological forms of words, or high level visual or auditory representations of concepts or words. Semantic pointers are defined as N-dimensional vectors (typically *N* = 512 for the case of coding an entire lexicon of a particular language, Crawford et al., 2015). The neural state representing a semantic pointer is a specific neural activation pattern occurring within a *cortical SPA buffer*. SPA buffers consist of several neuron ensembles that each represent a subset of the N dimensions in the semantic pointer. Like ensembles, the input to buffers can change over time, allowing a SPA buffer to represent different semantic pointers depending on input. While neuron ensembles can be decoded into a real-valued vector, SPA buffers require an additional operation to decode. In order to determine the semantic pointer currently represented by the neural state of a cortical SPA buffer, the similarity of the neural state is calculated for all semantic pointers defined for the current neural model. This similarity is calculated using the *dot-product operation* (Stewart and Eliasmith, 2014). The dot product should be near 1 if the state matches the target semantic pointer, and near 0 for other semantic pointers. Thus, a useful way of characterizing the activity pattern within a cortical SPA buffer is by way of its similarity values with all currently defined semantic pointers. Throughout this work we will make extensive use of this kind of characterization for visualization purposes (e.g., see **Figures 2–11** in Sections Methods and Results of this paper).

Semantic pointers are also the vehicles for representing *actions*, e.g., whether the production of a word should be started (e.g., buffer A1 = ‘SPEAK’) or halted (e.g., buffer A2 = ‘HALT’). The neural states for these semantic pointers are activated at the level of a cortical task control buffer, which is closely connected with an action selection module. This buffer, together with the basal ganglia and thalamus complex, forms the *cortico-cortical action selection loop* (Stewart et al., 2010a,b).

Because it is non-trivial to generate an amount of semantic pointers for representing the concepts, words (i.e., lemmas and orthographic forms), and phonological forms of words for a specific natural language vocabulary, including a representation of the similarities of words at the concept (or semantic) and at the phonological-phonetic level, a *semantic pointer network module* constitutes a further part of the NEF. Here N words WORD_i, where *i* = 1, …N, are stored within three subnetworks of semantic pointers, e.g., WORD_1_CONCEPT = ‘Apple_Apfel’ is part of subnet “concepts,” WORD_1_LEMMA = ‘W_Apple’ is part of subnet “words,” and WORD_1_PHONOL = ‘St_E_pel’ is part of the subnet “phonological representations.” The nomenclature for the semantic pointers is given in Section Methods. Thus, this semantic pointer network module allows the specification of semantic pointers for words at different levels of representations (e.g., conceptual, orthographic, and phonological levels), as well as the specification of relations and similarities between semantic pointers at different representation levels (see Section Methods in this paper and see Section 5.2 in Crawford et al., 2015).

In the following sections we introduce our model for speech production and perception including speech monitoring and error-processing, and we present experimental results (i) modeling halting in production when distortions are evoked at semantic and phonological levels within the model and (ii) for simulating a picture naming and halt task (Slevc and Ferreira, 2006).

## Methods

### Architecture of the neural model

**Figure 1:**
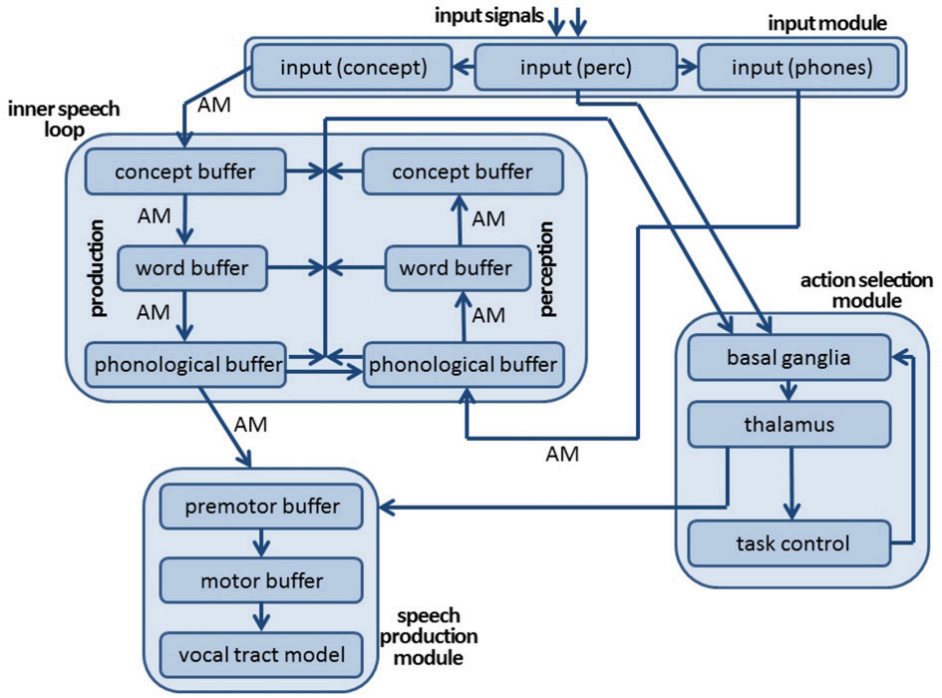
**Architecture of neural model of speech production and speech perception for modeling self-detection of speech errors and for modeling a picture naming and halt task**.

The architecture of the neural model comprises an *input module*, a production and a comprehension pathway forming the core of the *inner speech loop*, and an *action selection module* (Figure 1). The *speech production module*, shown in Figure 1, has been described in previous studies (Kröger et al., 2014, 2016; Senft et al., 2016) and is not included in the current study in order to keep simulation time low. The input module consists of three cortical SPA buffers: the perceptual, conceptual, and phonological input buffers (see Figure 1). The neural activations occurring at the level of the *perceptual input buffer* indicate the points in time when a visual or audio input signal appears. Semantic pointers are defined for representing these perceptual events, i.e., the points in time for the beginning and end of input in the visual and auditory domains. The neural signals generated in the perceptual input buffer are directly forwarded to the action selection module (Figure 1). In parallel, visual input information evokes a neural state within the *conceptual input buffer* and auditory input information evokes a neural state within the *phonemic input buffer*. Visual input is represented here in the form of conceptual semantic pointers, which encodes the meaning of the picture presented as the input signal at that point in time. Thus, specific visual processing is not currently included in our model. This omission of visual processing is justified, because subjects participating in the picture naming and halt experiment (Slevc and Ferreira, 2006; Experiment 1) undergo an initial familiarization procedure wherein they learn to associate 18 concrete English words (18 target words) with 18 concrete line drawings which are later presented in the task. Similarly, auditory processing was simplified by directly activating the phonological representation (i.e., a sequence of speech sounds or phones) associated with an acoustically presented distractor word (halt signal). This simplification too is justified, because the goal of this study is to test the *inner* speech loop (see Slevc and Ferreira, 2006, p. 518, Figure 2). The three buffers that make up the input module (like all other buffers in our model) are implemented as cortical SPA buffers (Stewart and Eliasmith, 2014), capable of representing 512 dimensional vectors (semantic pointers) using 50 neurons per dimension (25,600 neurons per buffer).

**Figure 2:**
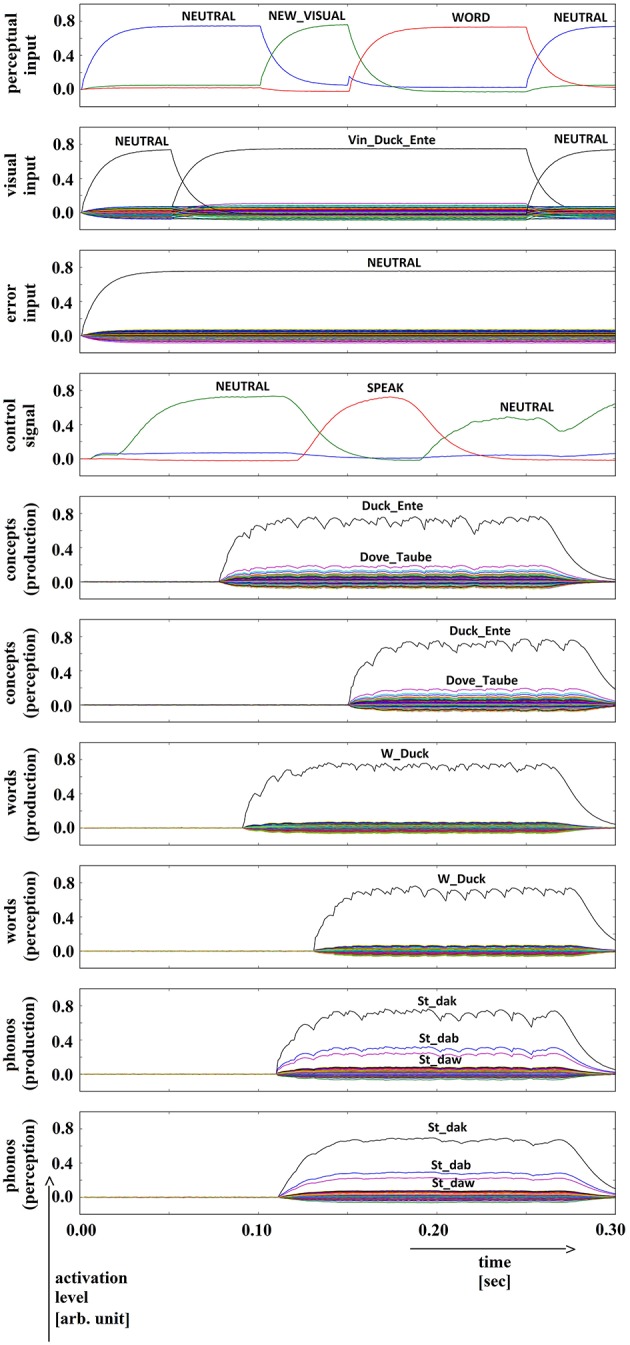
**Simulation of picture naming task without error stimulation; visual input (Vin): “duck,” no additional input; phonological form: ‘St_dak’**. Rows indicate neural activation levels of different cortical SPA buffers over time. Row 1: perceptual input buffer, row 2: conceptual input buffer (visual input ‘Vin_…’ is directly converted in a concept representation), row 3: error input buffer (not indicated in Figure 1), row 4: task control buffer, rows 5–10: cortical buffers for concepts, words, and phonological forms within production and perception/comprehension pathway of inner speech loop. In row 2, row 3, and rows 5–10, the activation levels of all 90 semantic pointers are displayed. Only the semantic pointers with the highest activation levels are labeled by text.

The *inner speech loop* consists of six *cortical SPA buffers*, representing the *conceptual, word*, and *phonological state* of a currently activated word. Within these six cortical SPA buffers (three for the production pathway and three for the perception pathway, see Figure 1), only neural states that represent semantic pointers of concept, word, or phonological forms of already learned words can be activated. These semantic pointers are stored as vectors within a portion of our neural model called the *mental lexicon module*. The concepts, words, and phonological forms stored in that module are listed in Appendix A. During picture naming, the neural state activated in the concept input buffer (i.e., the concept corresponding to the target word presented visually) directly co-activates concept-level, word-(lemma)-level, and phonological-level neural states for the target word in the *production pathway* (Figure 1). Subsequently, the phonological neural state of the production pathway co-activates a phonological, word, and perceptual neural state within the *comprehension pathway* in order to allow self-perception and self-monitoring. If, in addition, external speech (produced not by the model itself but by an interlocutor) is presented acoustically, then the phonological input representation, i.e., phonological representation of an external acoustically presented word (activating the phone input buffer within the input module of our model, see Figure 1), also co-activates the phonological, word, and conceptual SPA buffers of the perception pathway of the inner speech loop (the arrow from input module to inner speech loop in Figure 1). This externally-elicited activation interferes with the activation in the comprehension pathway that stems from the current state of the phonological component of the production pathway (see left-to-right arrow, also called a “shortcut” between both phonological buffers in Figure 1). The direct co-activation of related conceptual, word, and phonological states within both the production and perception pathways of the model is implemented using four (hetero-)associative memories (Voelker et al., 2014), labeled as AM in Figure 1. The associations stored in these four associative memories are considered to be part of the mental lexicon. For example, for the concept coded by the semantic pointer ‘Apple_Apfel’, the semantic pointer ‘W_apple’ is the associated representation at the word (lemma) level and the semantic pointer ‘ST_E_pel’ is the associated representation at the phonological level (see also Appendix A; concept pointers like ‘Apple_Apfel’ are written in two languages, because a concept is not necessarily language specific; the word representation for apple is labeled as ‘W_Apple’; phonological forms are given as phonetic-phonological transcriptions (e.g., ‘ST_E_pel’ for “apple”). Within these transcriptions, syllables are separated by an underline and the most stressed syllable within a word is marked by the prefix ‘St_’; the transcriptions in part follow SAMPA notation, SAMPA, 2005).

From a functional viewpoint, the neural model presented here is designed for (i) self-detection of speech errors occurring during word production by self-monitoring, and for (ii) realizing a picture naming and halt task, which requires the self-monitoring component in order to compare self-produced target words to externally produced distractor words. Consequently, the *action selection module* used in our model is primarily designed for doing self-monitoring and, in particular, for evaluating the degree of similarity between the neural states active in the production buffers to those active in the comprehension buffers at concept, word, and phonological levels (see arrow from inner speech loop to action selection module in Figure 1). In addition, the neural states that are currently active in the perceptual input buffer are fed to the action selection module in order to identify the points in time at which the comparison of production and perception neural states needs to be carried out in order to activate a ‘HALT’ action.

The similarity values representing concept, word, and phonological levels can be calculated as dot products (see Section Introduction). Thus, dot products are used here for calculating utility values Ui for actions Ai (*i* = 1, …, M) at the level of the *basal ganglia*. The action Ai exhibiting the highest utility value Ui is selected by the *thalamus* component of the action selection module (Stewart et al., 2010a; Eliasmith, 2013; Stewart and Eliasmith, 2014). The neural implementation of action selection relies on the interacting dynamics of excitatory AMPA connections and inhibitory GABA connections between different parts of the basal ganglia (i.e., striatum, substantia nigra, and globus pallidus externus/internus). Moreover, a detailed realization of the cortico-cortical loop including basal ganglia and thalamus has been implemented (Stewart et al., 2010b). It should be noted that the detailed modeling of post-synaptic time constants at cortical levels as well as at the level of the basal ganglia thalamus action selection module leads to a typical time interval of around 50 ms for action selection, also called the “cognitive cycle time” (Anderson et al., 2004; Stewart et al., 2010b).

**Figure 3:**
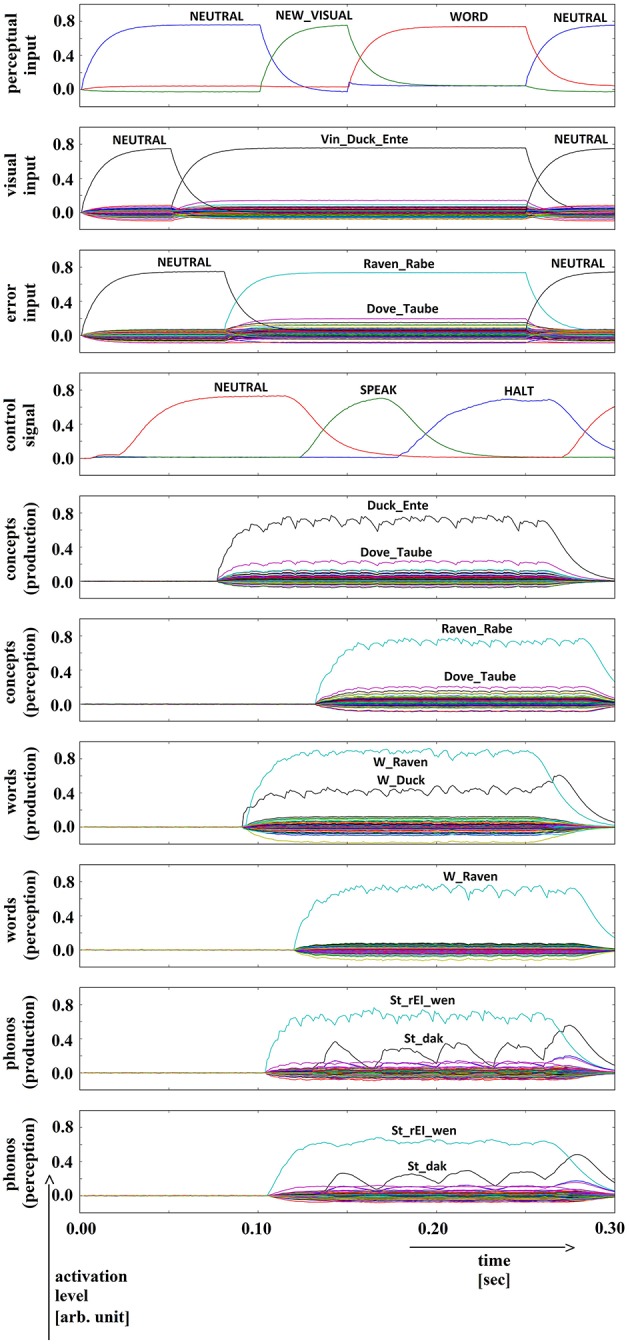
**Simulation of picture naming task with stimulation of a semantic (conceptual) speech error; visual input (Vin): “duck,” error input (conceptual): “raven”; phonological forms: ‘St_dak’, ‘St_rEI_wen’**. Rows indicate neural activation levels of different cortical SPA buffers over time (see Figure 2).

**Figure 4:**
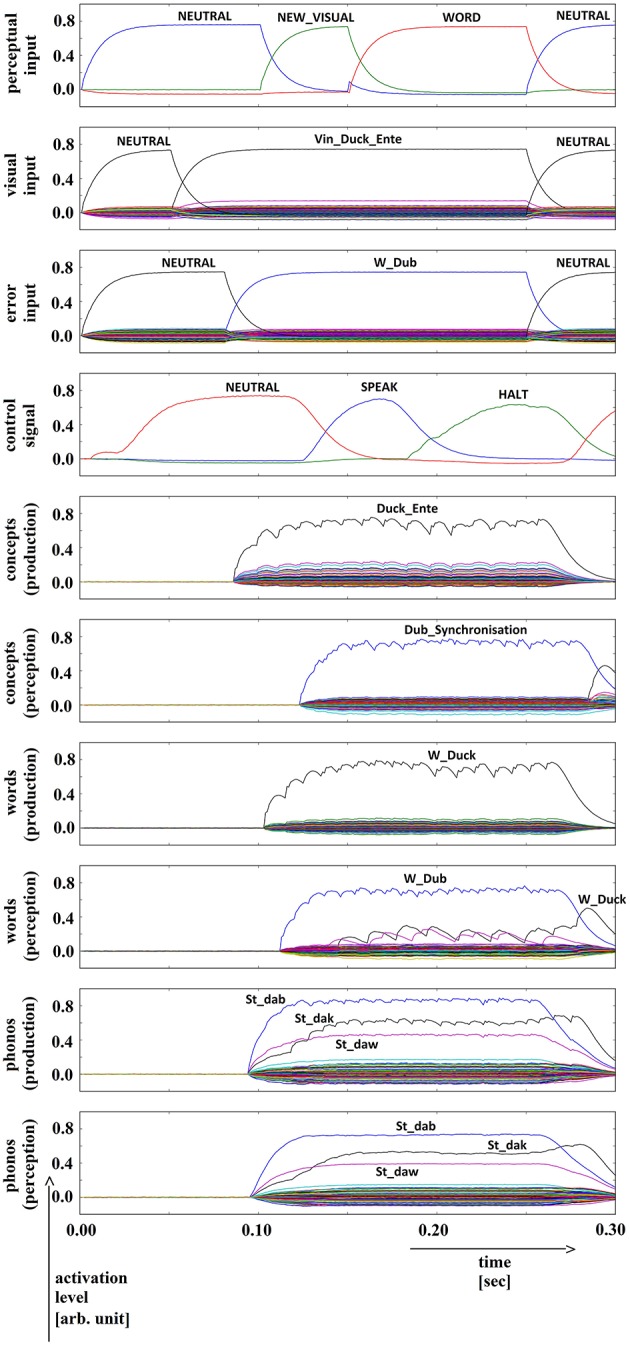
**Simulation of picture naming task with stimulation of a phonological speech error; visual input (Vin): “duck,” error (word) input: “dub”; phonological forms: ‘St_dak’, ‘St_dab’**. Rows indicate neural activation levels of different cortical SPA buffers over time (see Figure 2).

**Figure 5:**
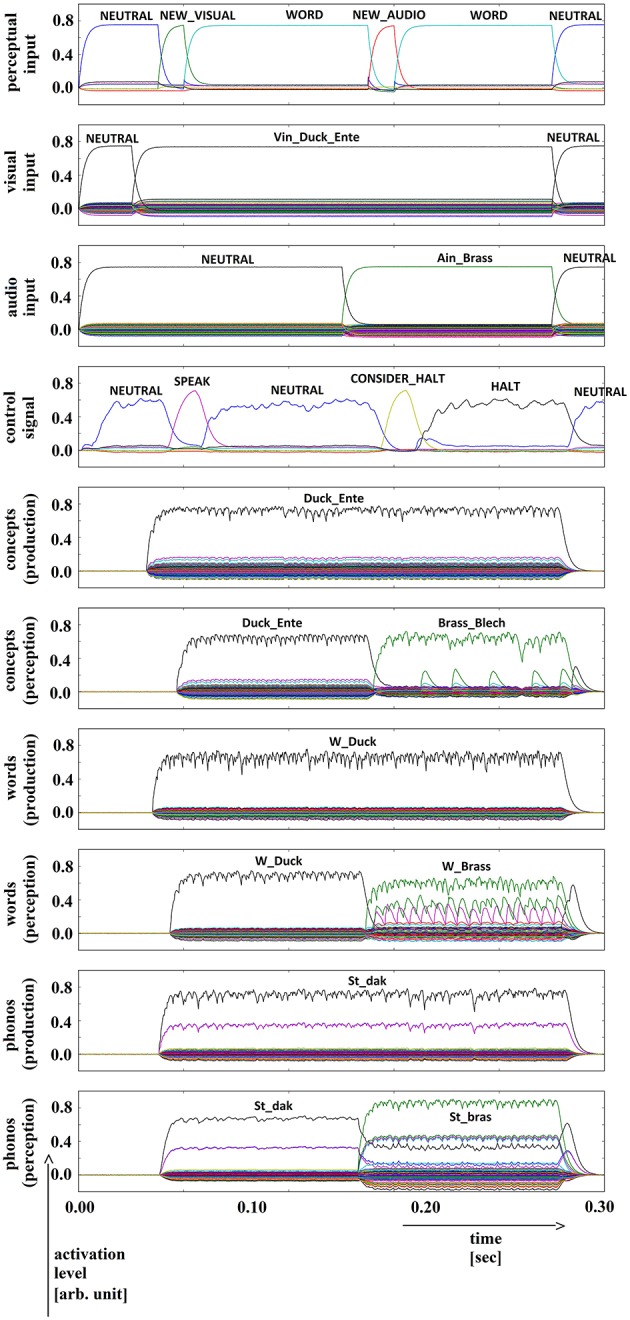
**Simulation of picture naming and halt task for dissimilar inputs; visual input (Vin): “duck,” auditory input (Ain): “brass”; phonological forms: ‘St_dak’, ‘St_bras’**. Rows indicate neural activation levels of different cortical SPA buffers over time (see Figure 2 with exception of row 3). Row 3: phone input buffer (audio input ‘Ain_…’ is directly converted in a phonological representation).

**Figure 6:**
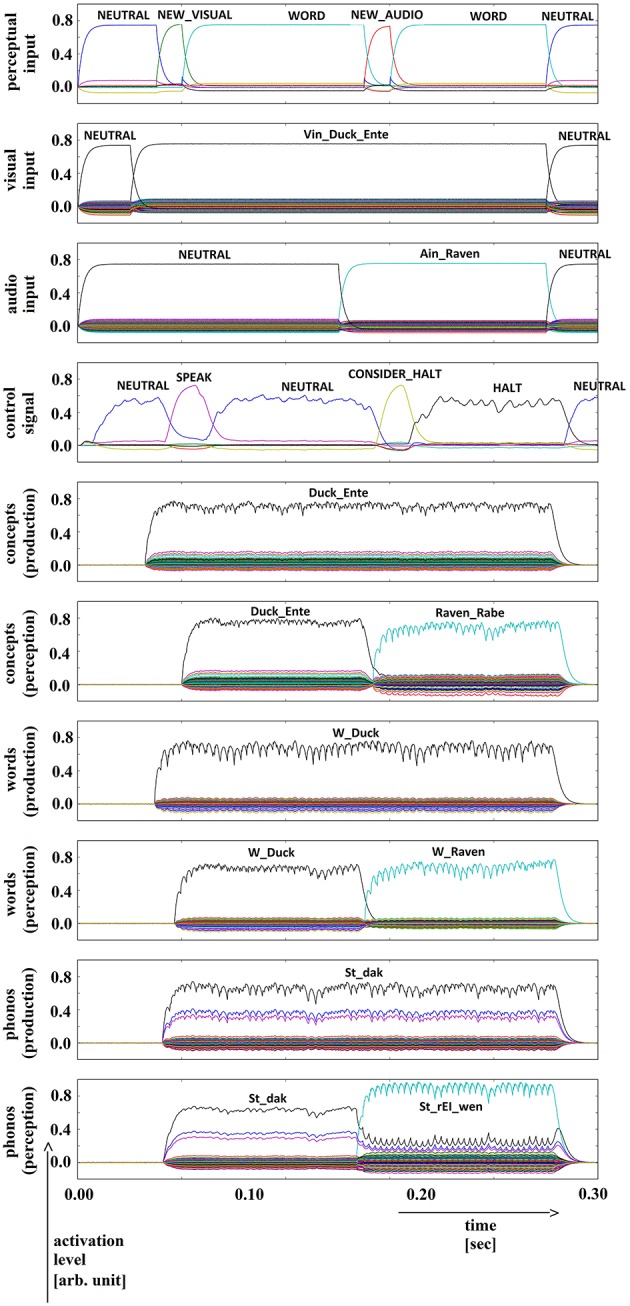
**Simulation of picture naming and halt task for semantically similar inputs; visual input (Vin): “duck,” auditory input (Ain): “raven”; phonological forms: ‘St_dak’, ‘St_rEI_wen’**. Rows indicate neural activation levels of different cortical SPA buffers over time (see Figure 5).

**Figure 7:**
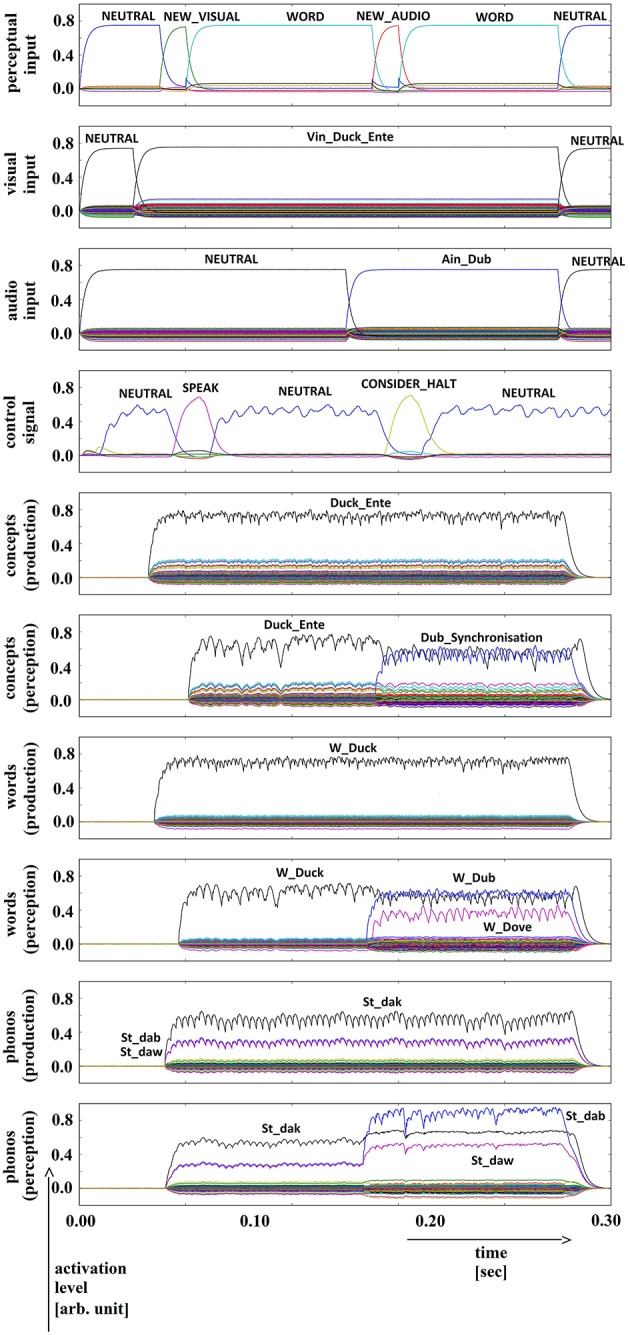
**Simulation of picture naming and halt task for phonologically similar inputs; visual input (Vin): “duck,” auditory input (Ain): “dub”; phonological forms: ‘St_dak’, ‘St_dab’**. Rows indicate neural activation levels of different cortical SPA buffers over time (see Figure 5).

**Figure 8:**
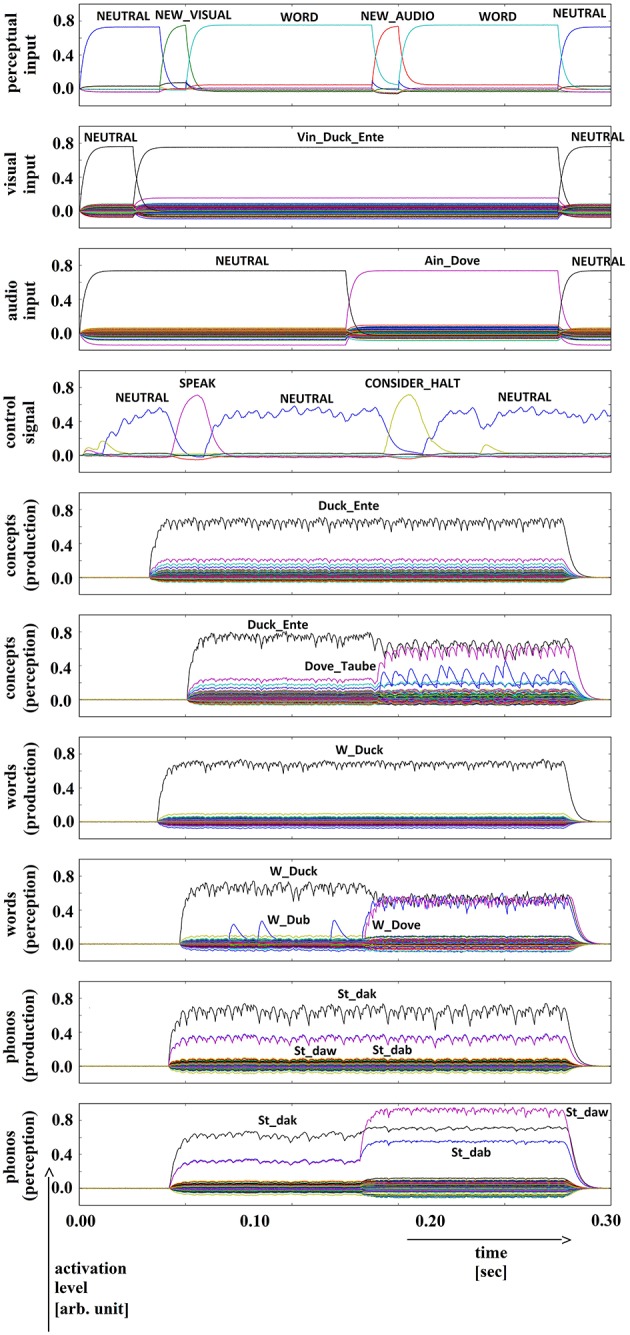
**Simulation of picture naming and halt task for semantically and phonologically dissimilar inputs; visual input (Vin): “duck,” auditory input (Ain): “dove”; phonological forms: ‘St_dak’, ‘St_daw’**. Rows indicate neural activation levels of different cortical SPA buffers over time (see Figure 5).

**Figure 9:**
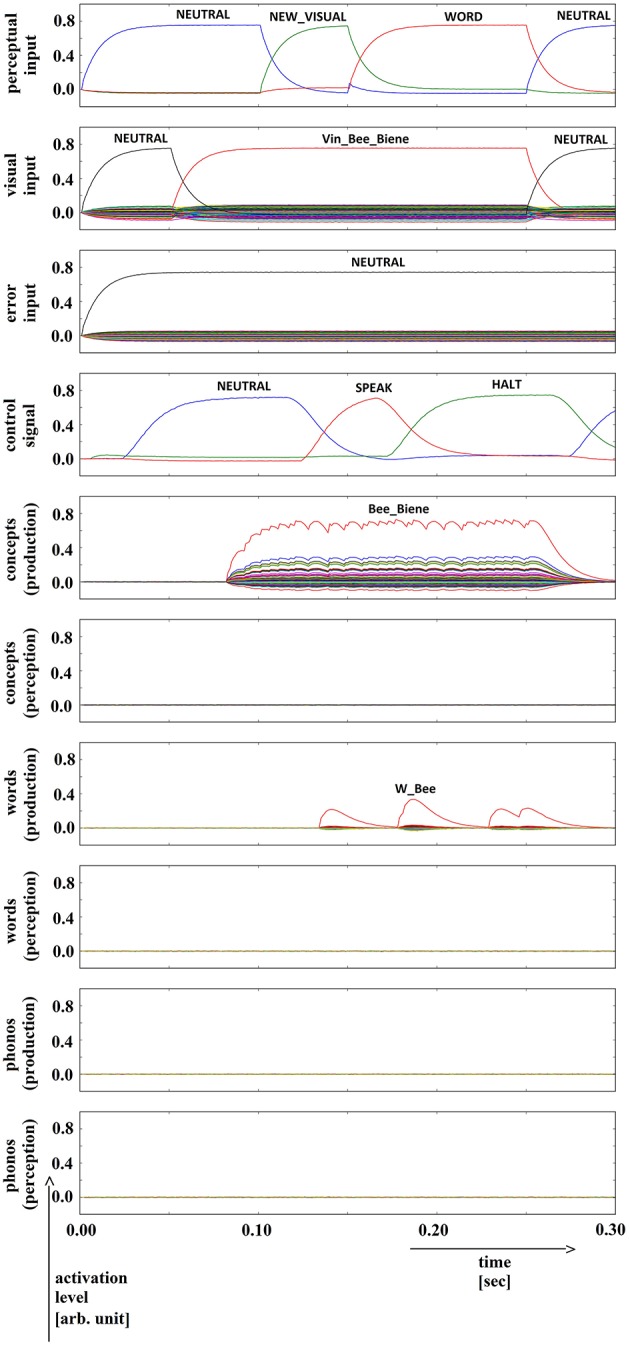
**Simulation of picture naming task without error stimulation for the rare event: no production signal (phonological level); visual input (Vin): “bee,” no additional input**. Rows indicate neural activation levels of different cortical SPA buffers over time (see Figure 2).

**Figure 10:**
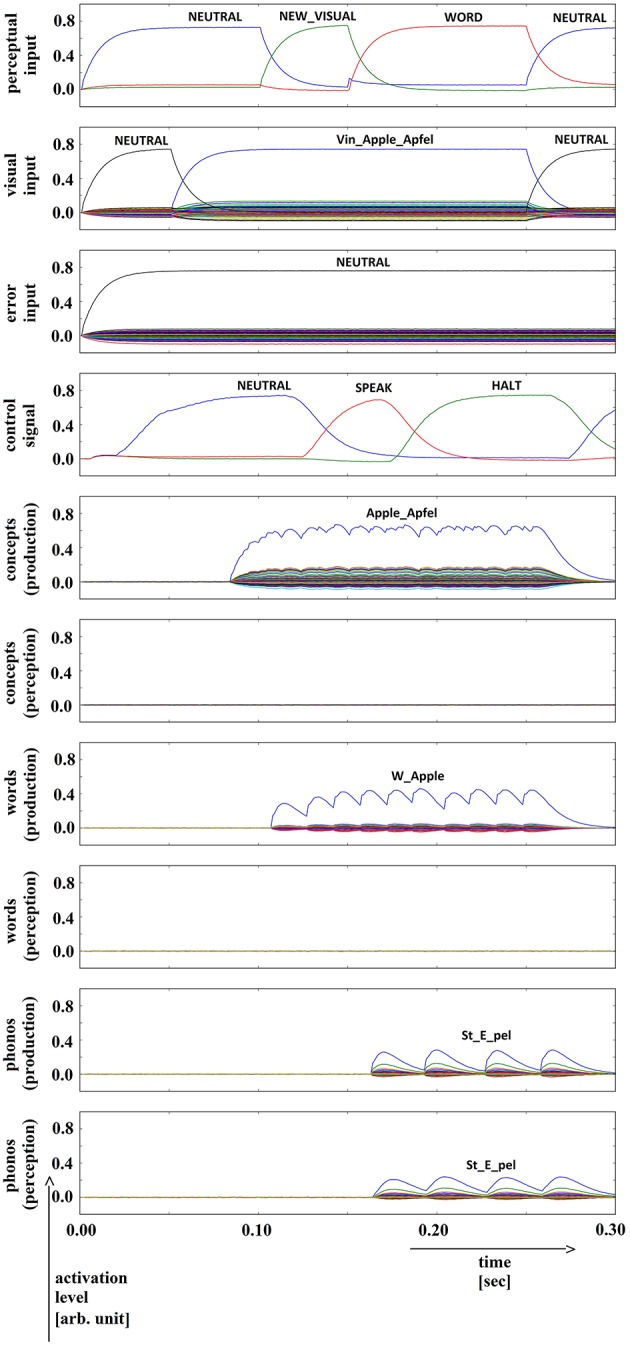
**Simulation of picture naming task without error stimulation for the rare event: weak production signal (phonological level); visual input (Vin): “apple,” no additional input**. Rows indicate neural activation levels of different cortical SPA buffers over time (see Figure 2).

**Figure 11:**
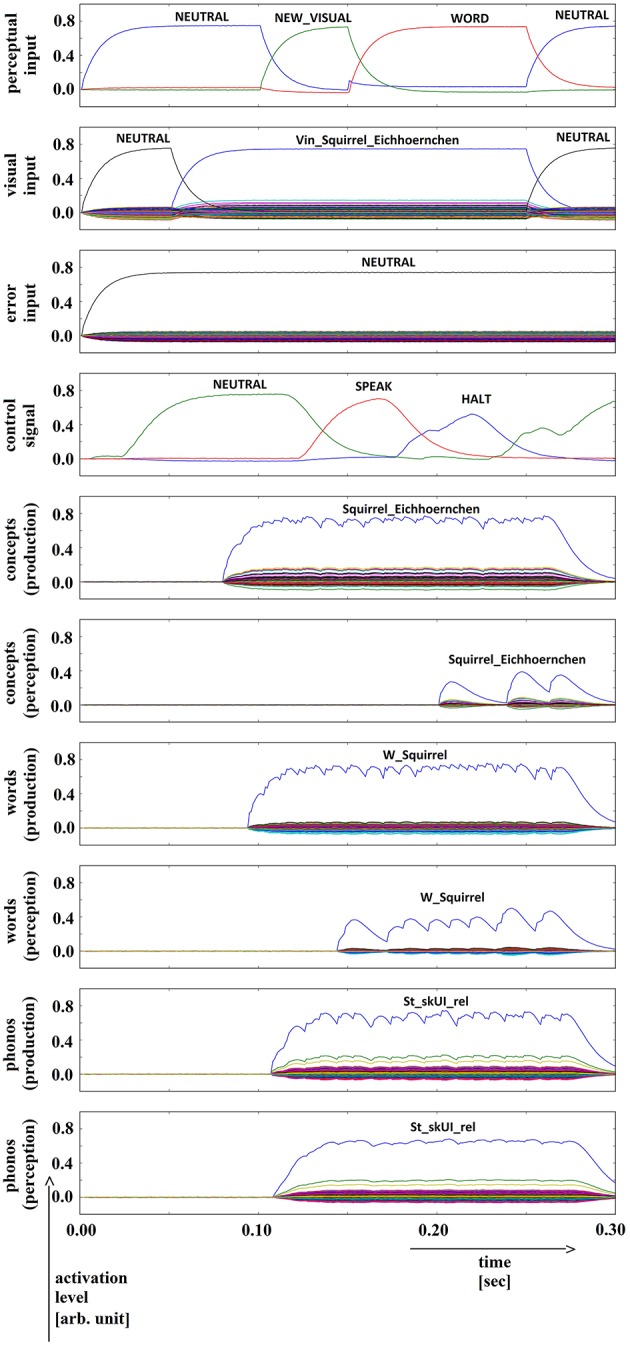
**Simulation of picture naming task without error stimulation for the rare event: weak signals in perception pathway (here: concept level) leading to (weak) halt signal; visual input (Vin): “squirrel,” no additional input**. Rows indicate neural activation levels of different cortical SPA buffers over time (see Figure 2).

The basic actions which can be selected in our model are A1 = ‘NEUTRAL’ (do nothing), A2 = ‘SPEAK’, A3 = ‘CONSIDER_HALT’, and A4 = ‘HALT’. If one of these actions becomes chosen, its semantic pointer similarity value approaches 1 within the time course of the neural activation patterns of the *task control cortical SPA buffer* within the action selection module (Figure 1; see row 4 in Figures 2–**11** below). Action selection works as follows: all dot products are continuously evaluated in order to estimate the utility values U2(t), U3(t), and U4(t) for the if-statements (ii) to (iv). Specifically,

U2(t) = DOT_PROD(perceptual_input_buffer, ‘NEW_VISUAL’)
U3(t) = DOT_PROD(perceptual_input_buffer, ‘NEW_AUDIO’)
U4(t) = DOT_PROD(perceptual_input_buffer, ‘WORD’) −
              DOT_PROD(concept_buff_perc, WORD_i_CONCEPT) +
              DOT_PROD(word_buff_perc, WORD_i_LEMMA) +
              DOT_PROD(phonol_buff_perc, WORD_i_PHONOL)

- if all utility values Ui(t) < 0.25 (where Ui(t) ranges between 0 and 1)
  then: select action ‘NEUTRAL’ (i.e., do nothing);
- if U2(t) is highest utility value currently
  then: select action A(t) = ‘SPEAK’;
- if U3(t) is highest utility value currently
  then: select action A(t) = ‘CONSIDER_HALT’;
- if U4(t) is highest utility value currently
  then: select action A(t) = ‘HALT’;

Thus, action selection mainly leads to ‘NEUTRAL’ if no dot product is above 0.25 (if-statement i). If a new word is activated by a visual signal at the concept input buffer, the ‘SPEAK’ action will always be chosen (if-statement ii). The semantic pointers ‘NEW_VISUAL’ indicates the beginning of a visual presentation of a new word during the time interval ‘WORD’. If an external audio signal is presented quickly after the activation of ‘SPEAK’ (as happens in the picture naming and halt tasks) then ‘CONSIDER_HALT’ will be activated (if-statement iii). In addition, if a word is clearly activated within all buffers of the inner speech loop (which is always the case after the ‘NEW_VISUAL’ input appears) the ‘HALT’ action could be chosen if the difference between semantic pointers activated in the production (i.e., WORD_i_CONCEPT, WORD_i_LEMMA, and WORD_i_PHONOL) and perception/comprehension pathways (i.e., current neural activation pattern within cortical SPA buffers concept_buff_perc, word_buff_perc, and phonol_buff_perc) is small at each of the concept, word and phonological levels (if-statement iv). If this difference is large, then utility value U4 will be low, leading to no activation of the ‘HALT’ action.

In our model, action selection indirectly leads to an activation of a go-signal for the speech production module (see the arrow from action selection to speech production in Figure 1). The go-signal will be activated if the ‘SPEAK’ action is selected and if no ‘HALT’ action becomes activated during the next 100 ms. The *speech production module* consists of a premotor buffer for representing the motor plan of the word that is currently activated in the phonological component of the inner speech loop (see the arrow between the inner speech loop and speech production module in Figure 1), a motor buffer for representing the muscle activation patterns of the speech articulators, and the (external) vocal tract model for representing the articulator movements and for generating the acoustic speech signal (assumed to represent the M1 cortical area; for the separation of motor planning and execution see Kröger and Cao, 2015 and Kröger et al., 2016). The production model allows primary motor activation only in the case of an active go-signal and if a motor plan is activated in the premotor buffer of the speech production module, which can only be the case if a clear and strong activation of a phonological form occurs in the phonological buffer within the production pathway of the inner speech loop. In order to keep simulation times low, the production module is not included in the simulations described in this paper. The neural model developed here for the simulations described below was programmed in Nengo (Bekolay et al., 2014).

### Network implementation of the mental lexicon

It has been mentioned above that the neural states activated in the concept, word, and phonological buffers within the inner speech loop are neural activation patterns equivalent to or represented by semantic pointers. These semantic pointers are stored as vectors within the mental lexicon module of our model (not shown in Figure 1). The vector representations and the neural states associated with these pointers are assumed to have been learned during speech and language acquisition. Because learning is beyond the scope of this paper, the collection of semantic pointers making up the mental lexicon is predefined in our neural model. This is realized by using *semantic pointer networks*, which define not only the number of semantic pointers but also the relations between them (e.g., relation “is a” in “apple is a fruit”; Eliasmith, 2013; Blouw et al., 2015; Crawford et al., 2015). Semantic pointer networks should not be confused with neural networks; rather, semantic pointer networks can be thought of as a way of representing a knowledge base, which can be implemented or realized by a spiking neural network using NEF methods. Before running a simulation, the semantic pointer network is generated for a pre-defined natural language vocabulary (see Appendix A).

In the case of the mental lexicon, the semantic pointer network needs to be subdivided into subnetworks for *concepts, deep concepts, words, phonological forms, deep phonological forms, visual input, and auditory input* (see Appendix A). All semantic pointers defined in each subnetwork and all relations between semantic pointers needed in each subnetwork and between different subnetworks are predefined and then used in the simulation in order to (i) generate a *vector representation* for each semantic pointer and to (ii) generate an *associated neural state* (neural activation pattern) for each semantic pointer. The subnetworks including all labels for semantic pointers and their relations are listed in Appendices A1–A5. While the subnetworks for concepts, words and phonological forms consist of 90 items each in the case of our vocabulary, the deep_concept and deep_phonological networks contain the semantic pointers needed to specify the relations between specific concepts and specific phonological forms.

Our neural model also requires subnetworks for visual representations of concepts and for auditory representations of words. In our experimental scenario, visual images are closely related to concepts, and aural signals are closely related to phonological forms. Each of these subnetworks contains 90 items, each of which corresponds directly to one of the words defined in the subnetworks for concepts, words, and phonological forms. The semantic pointers for visuals are labeled with the prefix ‘V_’ (e.g., ‘V_Apple_Apfel’) and the semantic pointers for aural signals are labeled with the prefix ‘A_’ (e.g., ‘A_apple’). In addition, semantic pointers are defined for visual and auditory input representations. The pointers within these subnetworks are labeled with an initial ‘Vin_’ or ‘Ain_’ respectively.

### Word corpus

Eighteen different input or target words are used in both simulation experiments (listed in the first column of Table 1). In Experiment 1, semantically similar distractor word activations are added at the word level and phonologically similar distractor word activations are added at the phonological level. These distractor words are listed in columns 2 and 3 of Table 1. In Experiment 2, the 18 visually presented target words are combined with four different auditory input words (i.e., stop signal or distractor words, cf. Slevc and Ferreira, 2006). These distractor words are (i) semantically similar, (ii) phonologically similar, (iii) semantically and phonologically similar, or (iv) semantically and phonologically dissimilar words in relation to their corresponding target word (see columns 2–5 in Table 1). The resulting 90 words for picture naming and/or for distortion (distractor or stop signal words) are collected into in a semantic pointer network (see Appendix A).

**Table 1:** **Words used as target words (column 1) or as distractor/stop signal words (columns 2–5) in the simulation experiments**.

**Picture name (target word) (visual input)**	**Semantically similar word (auditory input)**	**Phonologically similar word (auditory input)**	**Phonologically and semantically similar word (auditory input)**	**Dissimilar word (auditory input)**
Apple	Peach	Apathy	Apricot	Couch
Basket	Crib	Ban	Bag	Thirst
Bee	Spider	Beacon	Beetle	Flag
Bread	Donut	Brick	Bran	Nail
Camel	Pig	Cash	Calf	Bucket
Carrot	Spinach	Cast	Cabbage	Evening
Duck	Raven	Sub	Dove	Brass
Elephant	Moose	Elm	Elk	Stripe
Fly	Moth	Flu	Flea	Rake
Lamp	Candle	Landing	Lantern	Package
Peanut	Almond	Piano	Pecan	Dress
Rabbit	Beaver	Raft	Rat	Coffee
Snake	Eel	Snack	Snail	Fire
Spoon	Ladle	Sparkle	Spatula	Cable
Squirrel	Mole	Skate	Skunk	Chain
Train	Bus	Trophy	Trolley	Fox
Truck	Jeep	Trap	Tractor	Celery
Trumpet	Horn	Traffic	Trombone	Corner

*Word list is adapted from Damian and Martin (1999, Experiment 3)*.

### Experiment 1

#### Experiment 1a

We conducted 35 trials in which productions of each of the 18 target words were simulated (630 simulations in total). No distractors or stop signals were activated. Neural activation levels for different semantic pointers in different cortical SPA buffers are displayed in Figure 2 for a typical simulation trial. Here, the similarity between the neural activity and the most similar semantic pointers is given for 10 different cortical SPA buffers. It can be seen that all cortical SPA buffers for concept, word and phonological form reflect full activation for the target word semantic pointers during production as well as during perception (Figure 2, rows 5 to 10 for the concept/word/phonological form of “duck”). The perceptual input buffer (Figure 2, row 1) signals that a full activation of visual input ‘NEW_VISUAL’ occurs at approximately *t* = 100 ms. This input is held for a time interval of 150 ms until *t* = 250 ms (see activation of semantic pointer ‘WORD’ in Figure 2, row 1). The concept input buffer (Figure 2, row 2) indicates that the concept activation from visual input starts at about 50 ms, while full activation occurs at around 100 ms and holds for 150 ms until *t* = 250 ms. No error signal is generated (see Figure 2, row 3: activation of error input buffer is ‘NEUTRAL’). The semantic pointer activation within the task control buffer (Figure 2, row 4) indicates that the action ‘SPEAK’ will be activated at around *t* = 170 ms. This action results from the evoked perceptual input (i.e., we evoke the ‘NEW_VISUAL’ semantic pointer). The action selection module always activates the ‘SPEAK’ action if the neural activity pattern within the cortical task control buffer previously represented the ‘NEW_VISUAL’ semantic pointer (see Section Architecture of the Neural Model).

In the lower part of Figure 2 (rows 5–10) the levels for the semantic pointers activated at the concept, word, and phonological form levels are displayed for the production and perception pathways; these will be interpreted in the Results Section.

#### Experiment 1b

We simulated ten trials in which the model produces each of the 18 target words with some kind of distortion (column 1 in Table 1; 180 simulations in total). Two different types of distortions were introduced (5 trials each). (i) A “concept distortion” was introduced by activating a distractor word which was semantically similar to the target word (column 2 in Table 1) and by adding this activation in the word buffer of the production pathway (Figure 1). (ii) A “phonological distortion” was introduced by activating a distractor word which was phonologically similar to the target word (column 3 in Table 1) and by adding this activation to the phonological buffer in the inner speech loop (Figure 1).

The induction of a conceptual (or semantic) speech error in a picture naming task was simulated by adding a second concept buffer to the production pathway and by connecting the output of that second buffer (“side branch buffer”) together with the output of the original concept buffer (given in Figure 1) to the word buffer within the production pathway. The temporal activation pattern of this second concept buffer is displayed in the third row in Figure 3 (see error input buffer; in this example the concept activation for the distortion word “raven” is displayed). Thus, this “side branch production concept buffer” (not shown in Figure 1) is activated by a word which is semantically similar but not identical to the target word (“duck”).

A similar process was used to induce phonological speech errors. Specifically, a second word-level buffer was added to the production pathway of the inner speech loop, and was connected to the phonological buffer of the production pathway. This new word-level buffer is activated by a word which is phonologically similar but not identical to the target word. This leads to strong activation of the distortion word in the phonological buffer of the production pathway, which is then propagated to all levels of the perception pathway. The temporal activation pattern of the second word buffer is displayed in the third row in Figure 4 (error input buffer; displays the word activation for the distortion word “dub”).

### Experiment 2

Five trials of 72 word combinations (i.e., 18 target words and 4 × 18 = 72 distractor words) were used in the picture naming and halt task. Target words were activated by picture naming (column 1 of Table 1). Distractor words were activated after a short delay. Four different categories of distractor words were used: (i) words semantically similar to the target word (column 2 of Table 1), (ii) words phonologically similar to the target word (column 3 of Table 1), (iii) words semantically and phonologically similar to the target word (column 4 of Table 1), and (iv) words dissimilar to the target word along both dimensions (column 5 of Table 1). The results of a simulation experiment for each of these four cases are displayed in Figures 5–8.

In all four simulations of the picture naming and halt task, visual input starts at about 100 ms and is fully activated at 150 ms. The audio input starts at about 500 ms and is fully activated at 550 ms. The perceptual input buffer signals the presentation of new visual or audio input (row 1 in Figures 5–8). The visual input signal is represented in the visual input buffer directly by its concept representation while the audio input is represented in the audio input buffer directly by its phonological representation (rows 2 and 3 in Figures 5–8).

### Source code of the model and the experiments

Nengo source code for this model can be downloaded at http://www.phonetik.phoniatrie.rwth-aachen.de/bkroeger/documents/ipynb_InnerSpeechLoop.zip. This zip file includes 4 scripts in IPython notebook format, representing Experiment 1a, Experiment 1b with semantic distortions, Experiment 1b with phonological distortions, and Experiment 2 (picture naming with halts). This source code requires Nengo (version 2.0; Bekolay et al., 2014), which can be downloaded at http://www.nengo.ca/download.

## Results

### Experiment 1a

We simulated normal productions of the 18 target words listed in Table 1 (column 1) for 35 trials each (630 simulations in total). Semantic pointer activation patterns for a typical simulation trial are given if Figure 2. We can see that the temporal succession of word semantic pointer activations at concept, word and phonological levels for the production and perception pathways of the inner speech loop depends only on the signal activation in the visual (or conceptual) input buffer. Furthermore, it can be seen from Figure 2 that the time delay between activation of the concept buffer in the production pathway and the activation of the concept buffer in the perception pathway is only about 70 ms. This results from the direct associations realized by the associative memories, occurring between all six cortical SPA buffers in the inner speech loop. Thus, in our model the ‘SPEAK’ action (i.e., the action for starting the production and articulation process) is activated once the word has been propagated through all buffers of the inner speech loop, from production to perception. This time interval overlaps with the time interval for the input semantic pointer ‘WORD’.

It can further be seen that in the case of normal production (i.e., no distractor or error signal activation) the activities in all six cortical SPA buffers represent the (same) target word (“duck” in the case of the example given in Figure 2). At the phonological level, a weak co-activation of phonologically similar words also occurs. This results from the fact that the target word “duck” is phonologically (as well as phonetically and acoustically) similar to the words “dub” and “dove” (see also the relations for these words in Appendix A3; the semantic pointer of the deep phonological subnetwork, ‘PSt_da’, is similar for these words). Thus, this co-activation directly results from the close relation of the phonological forms of these three words; i.e., it directly results from the organization of the mental lexicon at the level of the semantic pointer network (see Section Network Implementation of the Mental Lexico). Similarly, at the concept level of the perception pathway we can see a weak co-activation of semantically similar words (i.e., “dove” and “raven”; see Appendix A1: “duck,” “dove,” and “raven” all contain the deep concept ‘Bird_Vogel’, making the conceptual semantic pointers of these words similar to one another). This co-activation of concepts once again results from the organization of the mental lexicon at the conceptual level.

In summary, for all 630 simulation trials, at no point in time were any of the conceptual, word or phonological buffers found to represent a semantic pointer for any word different from the target word. The phonological buffer within the production pathway was not activated at all in four trials (0.6% of all trials; see Figure 9 for an example, and see Table 2). A very weak and temporally discontinuous target word activation within the production pathway phonological buffer occurred in nine trials (1.4% of all trials; see Figure 10 for an example, and see Table 2). In all such cases, the neural connections linking the concept buffer to the word buffer and those linking the word buffer to the phonological buffer did not relay the neural activation patterns correctly, which seems to be the cause for these rare events. The ‘HALT’ action in the cortical task control buffer was activated in all of these instances. This action leads to a complete stop of production, which is the correct choice if no phonological representation is provided by the inner speech loop as input to the speech production module (see Figure 1). In other trials, weak activation of at least one of the three buffers occurred in the comprehension pathway, while neural activation within production pathway SPA buffers was normal (23 trials, 3.7%; see Figure 11 for an example, and see Table 2). This weak activation may lead to erroneous self-perception, and subsequently the activation of a ‘HALT’ action at the level of the task control buffer. Here, the utility of the ‘HALT’ action is lower than in the previously described production pathway deficits (see Figure 10 vs. Figure 11) and does not lead to the generation of a stop signal, but may lead to delayed activation of speech execution.

**Table 2:** **Percentage (and absolute number) of trials exhibiting “rare events” and generating “HALT” actions**.

**Rare event (630 total trials)**	**Not accumulated**	**Halt signals accumulated**
No production signal	0.6% (4)	0.6% (4)
Weak production signal	1.4% (9)	2.0% (13)
Erroneous self-perception	3.7% (23)	5.7% (36)

*Three cases can be separated: (i) no or (ii) weak production signal at level of phonological buffer; (iii) weak self-perception signals (i.e., weak signals in at least one of the perception buffers)*.

### Experiment 1b

The same neural model as in Experiment 1a (i.e., the model given in Figure 1) was used for Experiment 1b, with the addition of phonological or semantic distractor activations (phonological or semantic “distortions”) induced by the inner speech loop through “side branches” (see Section Experiment 1).

Semantic distractor activation (Figure 3): Since both concept buffers (normal and side branch) are connected to the word buffer in the production pathway, we get a strong activation of two words at this level (see Figure 3, row 7: activation of “raven” and “duck”; i.e., an *ambivalent neural activation pattern*). It can be seen from the example in Figure 3 that further activation of buffers in the inner speech loop leads to a strong activation of the distortion word at phonological, word and conceptual levels. The resulting difference in neural activation at the SPA buffers in the production and perception pathways is fed back to the action selection module and leads to a strong activation of the ‘HALT’ action at the level of the task control cortical buffer (see fourth row in Figure 3). Thus, the inner (semantic) speech error is clearly detected by the model itself.

Phonological distractor activation (Figure 4): In a manner similar to the semantic distractor activation above, here as well the disparity between representations in the production and perception pathways (rows 5–10 in Figure 4) is detected by the action selection module, which subsequently issues a ‘HALT’ action, signaling that the error has been detected.

In both the semantic distractor and phonological distractor cases, we consider two conditions with respect to the relative strength of the side-branch connections compared to the standard connections: (i) strong coupling, in which the side branch connections were twice as strong as the regular connections, and (ii) weak coupling, in which the side branch connections had the same strength as the regular connections. In each condition, five trials were simulated for each of the 18 target words (column 1 in Table 1) by adding a semantically (column 2 in Table 1) or phonologically (column 3 in Table 1) similar distortion word as side branch input. Results are given in Table 3. It can be seen from Table 3 that our model is capable of generating phonologically as well as semantically induced errors with a high rate. Semantically induced errors are detected with greater frequency than phonetically induced errors in both coupling conditions (Fisher's exact test: *p* < 0.001 in both cases).

**Table 3:** **Results of experiment 1b: Percentage (and absolute values) of evoked halts in the picture naming task with semantically or phonologically induced distortion words and different degrees of side branch coupling within the inner speech loop (90 trials per cell)**.

**Degree of coupling (90 total trials)**	**Semantically similar**	**Phonologically similar**
Strong	100.0% (90)	31.1% (28)
Weak	56.7% (51)	10.0% (9)

### Experiment 2

Five trials of 72 word combinations were used in the picture naming and halt task (18 target words induced by picture naming coupled with 18 semantically similar, phonologically similar, semantically and phonologically similar, and dissimilar distractor words for a total of 72 combinations; see Section Experiment 2).

For every word combination, ‘SPEAK’ and ‘CONSIDER_HALT’ actions are activated by the action selection module (see row 4 in Figures 5–8). These actions result from the input signals ‘NEW_VISUAL’ and ‘NEW_AUDIO’ forwarded from the perceptual input buffer. The ‘SPEAK’ action is activated upon presentation of visual input and the ‘CONSIDER_HALT’ action is activated upon presentation of audio input. For some distractor words, the ‘CONSIDER_HALT’ action is directly followed by a ‘HALT’ action (Figures 5, 6), while no such action occurs in other cases, e.g., if the similarity between the target and distractor words is too strong; see the if-then rules defined for the action selection system of our model (Section Architecture of the Neural Model). Rows 5–10 in Figures 5–8 show the semantic pointer activation levels for the conceptual, word and phonological buffers within production and comprehension pathways of the inner speech loop.

It is interesting to see that the phonological input signal to the comprehension pathway always overrides the input signal from the production pathway (i.e., the inner speech signal) in the case of phonologically different word pairs (Figures 5, 6). This is not the case for phonologically similar word pairs which may prevent speech error detection in the case of phonologically similar distractor words (Figures 7, 8 and see Section Discussion in this paper).

In summary, the percentage and absolute number of halted trials is given in Table 4 for each of the four types of target/distractor word combinations and for three different degrees of coupling strength of auditory input to the phonological buffer of the perception pathway. Strong, medium, or weak coupling means that the strength of the connection between the auditory input and phonological buffer is three, two or one times the strength of all other connections, respectively. Each cell in Table 4 results from 5 × 18 = 90 trials (1080 simulations in total).

**Table 4:** **Percentage (and absolute number) of evoked halts in the picture naming task for different types of target/distractor word combinations and for different degrees of coupling of auditory input with the inner speech loop**.

**Degree of coupling (90 total trials)**	**Semantically similar**	**Phonologically similar**	**Semantically and phonologically similar**	**Dissimilar**
Strong	60.0% (54)	0% (0)	0% (0)	96.7% (87)
Medium	21.1% (19)	0% (0)	0% (0)	68.9% (62)
Weak	5.6% (5)	1.1% (1)	0% (0)	14.4% (13)

It can be seen from Table 4 that the percentage of halted productions is nearly zero when the target and distractor are phonologically similar, while this is not the case for dissimilar or (only) semantically similar word combinations. Furthermore, semantic vs. dissimilar word pairs result in a significant difference in all three cases of degree of coupling (Fisher's exact test: *p* < 0.001 in all three cases).

## Discussion

The main goal of this paper is to develop a spiking neuron model of speech processing at multiple cognitive levels, mainly for word and phonological form selection and monitoring at the level of the mental lexicon. Because temporal aspects can be modeled in the Neural Engineering Framework and because this model—as with all spiking neuron models—generates variations from trial to trial at the level of neural states and their processing, it was possible to test the quality of this model by (i) checking the “natural” occurrence of speech errors, by (ii) checking whether the model is capable of generating speech errors if we evoke ambivalent neural states at different cognitive levels within speech production by including “side branches,” and by (iii) comparing the simulation results of a picture naming and halt task with human data. Our model and simulation results showing error-production and picture naming and halt performance reinforce the assumption that an inner speech loop exists. That is, the assumption of interacting production and perception pathways reinforce the existence of an inner speech monitor that compares production and perception related neural states at different cognitive levels within the inner speech loop. Thus, our spiking neuron model is a comprehensive cognitive approach for modeling and monitoring lexical access.

Because of the trial-to-trial variation of our neural model, “rare events” occurred at the cognitive levels of neural activation, which resulted in a stop of word production in 2.0% of 630 trials of normal word production (Experiment 1a; see Table 2). These rare events resulted from weak neural activations in the production buffers representing word and/or phonological states within the speech production pathway of the inner speech loop. In a further 3.7% of the trials weak or ambiguous activations occurred in the (self-)perception pathway (see Table 2). It is hypothesized that these further “rare events” could lead to delayed execution of the intended target word. It should be noted that we were not able to generate speech errors in form of word substitutions. This may result from the fact that the neural activation level for competitive phonologically or semantically similar words is stronger in humans than in the model developed here. But as was stated in Sections Methods and Results, our model already generates co-activations of words if these words are semantically and/or phonologically similar to the target word. Because speech errors in the form of word substitutions are very rare (around 0.1% in normal spontaneous speech; 166 word, syllable and segment errors were identified in 3000 to 5000 words spoken by 34 subjects in Garnham et al., 1981) it could be necessary to do more simulations (e.g., around 10,000 word productions) in order to generate roughly 10 substitutions. This error-scarcity is one of the reasons that researchers evoke speech errors by using specific experimental paradigms (communication scenarios) in behavioral experiments (Frisch and Wright, 2002; Goldstein et al., 2007).

In order to generate speech errors in our model, “side branches” were added within the production pathway of the inner speech loop (Experiment 1b, and see descriptions of the side branches in Sections Methods and Results). These side branches result in an increase in activation of competitive words which are semantically or phonologically similar to the target word. In both cases (adding neural activations for semantically or phonologically similar words) the model is capable of generating and detecting speech errors at a high rate (between 10 and 100%, see Table 3). The results of this experiment suggest that speech errors occur more often for semantically similar words than phonologically similar words (factor 3 to 6, see Table 3).

Because the introduction of “side branches” is a modification of the production model (modeling a modification within the brain of the speaker), speech errors are induced in behavioral experiments by modifying the communication or production scenario (i.e., the experimental paradigm). One kind of “artificial” production scenario—not directly for evoking speech errors but to investigate the speech monitoring function—is the introduction of an acoustical stop signal in a picture naming task (Slevc and Ferreira, 2006). A simulation of this experimental paradigm using our neural model exhibited the behavioral patterns (similar to those found in human behavioral data) that dissimilar distractor words and semantically, but not phonologically, similar distractor words lead to a high rate of halting in the production of the target word (Table 4). In our neural model, this halting results from the detection of dissimilarities between the target and distractor words at the level of the inner speech loop and can be explained by assuming an inner speech monitor that is constantly comparing the neural states in the production and the perception pathways. This monitoring is assumed to be an important speech error detection mechanism in slips of the tongue and thus the picture naming and halting paradigm is a promising experiment for investigating speech (self-)monitoring and self-detection of speech errors (Hartsuiker and Kolk, 2001).

In the case of phonologically similar distractor words, human data give a halting rate of about 20% in comparison to a halting rate of around 40% for semantically similar or completely dissimilar distractor words (Slevc and Ferreira, 2006, p. 521). We were not able to reproduce the 20% result using our model, but as in the human data our model predicts that halting occurs significantly more often for dissimilar or semantically similar distractor words. The fact that we were not able to model that result more closely from a quantitative viewpoint may result from low magnitudes for the dot products for phonologically similar distractor to target words occurring within the production and perception pathway of the inner speech loop, which is fed back to the action selection module in order to allow the starting of a ‘HALT’ action. It could be a task for a future study to modify the thresholds of the utility values in the basal ganglia (see Section Architecture of the Neural Model), or to modify the semantic pointers generated for the simulation in order to obtain a better quantitative match with human data.

In summary, like humans, the model is capable of halting when the distractor is dissimilar or only semantically similar to the target word. The fact that the model does not halt on distractors that are phonologically similar to the target forms in the picture naming and halting paradigm may result from the fact that the evaluation of similarities of neural activations between production and perception pathways in the inner speech loop seems to under-emphasize differences at the phonological level in comparison to differences at the semantic level. But recall that our model includes an “inner loop shortcut” connecting the phonological buffer of the production pathway to that of the perception pathway. This shortcut could be the source of an assimilation of the phonological forms within the production and perception pathways and thus also be the source of the difficulty of detecting differences between phonological forms in the production and perception pathways. But this shortcut is inevitable, because it is the basis for self-monitoring of inner speech. Moreover, we have demonstrated that phonological errors can be induced and detected by our model when side-branches are used (see the results of Experiment 1b, Table 3).

Finally, it should be noted that the degree of similarity for phonologically and semantically similar word pairs is quantitatively comparable from the viewpoint of the implementation of these representations at the level of the mental lexicon. For all 18 word pairs (rows in Table 1) the combination of the target word with phonological or semantically similar words leads to the same deep representations at the level of concepts (Appendices A1, A2) as well as to the same deep representations at the level of phonological forms (Appendices A3, A4). Eighteen different deep representations occur in the case of the concept representations, but only 15 different deep representations occur in the case of the phonological representations for modeling the similarity with the 18 target words. Fifteen different deep representations occur in the case of the phonological forms because the same deep phonological representations are used for three target words. The remaining 7 deep representations for concepts describe higher level dependencies within the deep concept network itself, while the other 18 deep representations only describe deep concepts directly used in the concept network (Appendix A1).

## Conclusions

In this paper we have proposed a comprehensive spiking neuron model of the inner speech loop. This model includes a word production pathway starting from the conceptual level and ending with the phonological level, as well as a word perception (and comprehension) pathway starting from the phonological level and ending with the conceptual level. While this paper has focused on interactions between production and perception during inner speech, the proposed model also has the potential to be a sensible starting point for speech processing in general. In particular, a number of straightforward extensions suggest themselves. (i) A sentence-level module could be added in order to facilitate production and comprehension of whole utterances. This could be a good starting point for investigating face-to-face communication processes from a modeling perspective (Kröger et al., 2010, 2011). (ii) While this word processing model focuses on retrieving information from the mental lexicon (i.e., semantic and phonological knowledge repositories for words), it should be possible to add a lower level speech production module controlling an articulatory model, which could then generate acoustic speech signals (Kröger et al., 2011), as well as incorporate a lower level speech perception module in order to process acoustic signals and activate words or phonological forms directly from acoustic input (Kröger and Cao, 2015). In summary, a complete biologically informed model of speech processing can not only shed light on the neural processes of speech production and perception, but may also be a valuable starting point for solving open problems in speech synthesis (e.g., highly natural sounding speech, Zen et al., 2009) and speech recognition (e.g., speech recognition in noisy environments, Mattys et al., 2012).

## Author contributions

All authors contributed to software coding, writing, and correcting the manuscript. BK conducted the experiments.

### Conflict of interest statement

The authors declare that the research was conducted in the absence of any commercial or financial relationships that could be construed as a potential conflict of interest.

## Appendix

### Appendix A1: subnetwork for concepts (python dictionary)

i) First two lines: Relation types are listed here. ‘VisR’: Visual Impression related to a concept; ‘AudiR’: auditory impression of the word, representing the phonological form of the word; ‘OrthoR’: orthographic representation of the word; ‘PhonoR’: phonological representation of the word; ‘IsA’: concept is member of a group, defined by a subordinate concept.

ii) Concepts = {}: 90 concepts are listed as semantic pointers in the Python dictionary concepts = {}. Each semantic pointer, representing one concept is listed at the beginning of a new line. Relations are listed in the same line.

Concepts are defined by an English-German word pair, representing that concept (e.g., ‘Apple_Apfel’). Visual representations are directly related to concepts and include a prefix ‘V_’. Auditory, orthographic and phonological representations are related to a word, here to the English word, representing the concept.

   visR = ‘VisR’, audiR = ‘AudiR’, orthoR = ‘OthoR’, isA = ‘IsA’

Relation_types = [visR, audiR, orthoR, phonoR, isA]

concepts = {

‘Almond_Mandel’: [(visR, ‘V_Almond_Mandel’), (audiR, ‘A_Almond’), (orthoR, ‘W_Almond’), (phonoR, ‘St_El_mend’), (isA, ‘Nut_Nuss’)],

‘Apathy_Apathie’: [(visR, ‘V_Apathy_Apathie’), (audiR, ‘A_Apathy’), (orthoR, ‘W_Apathy’), (phonoR, ‘St_E_pe_si’)],

‘Apple_Apfel’: [(visR, ‘V_Apple_Apfel’), (audiR, ‘A_Apple’), (orthoR, ‘W_Apple’), (phonoR, ‘St_E_pel’), (isA, ‘Fruits_Obst’)],

…

‘Trumpet_Trompete’: [(visR, ‘V_Trumpet_Trompete’), (audiR, ‘A_Trumpet’), (orthoR, ‘W_Trumpet’), (phonoR, ‘St_trOm_pet’), (isA,

‘BrassWind_BlechblasInstr’)]

}

### Appendix A2: subnetwork for deep concepts (python dictionary)

i) First two lines: Relation type: ‘IsA’: concept is member of a group, defined by a subordinate concept.

ii) concepts_deep = {}: Deep Concepts are listed as semantic pointers in the Python dictionary concepts_deep = {}. Each semantic pointer, representing one deep concept is listed at the beginning of a new line. Relations are listed in the same line. Deep concepts will be used in concept dictionary (see Appendix A1)

   isA = ‘IsA’

Relation_types = [isA]

concepts_deep = {

# deep concepts, describing relations between concepts in concept-network:

‘Bin_Behaelter’: [(isA, ‘Object_Gegenstand’)],

‘Bird_Vogel’: [(isA, ‘Animal_Tier’)],

‘Bluebottle_Brummer’: [(isA, ‘Insect_Insekt’)],

…

‘Vegetables_Gemuese’: [(isA, ‘Food_Nahrung’)],

# deep concepts, describing relations within deep concept network:

‘ClovenHoofed_Paarhufer’: [(isA, ‘FourLeg_Vierbeiner’)],

‘FourLeg_Vierbeiner’: [(isA, ‘Animal_Tier’)],

‘Insect_Insekt’: [(isA, ‘Animal_Tier’)],

‘Vehicle_Fahrzeug’: [(isA, ‘Object_Gegenstand’)]

# basic level:

‘Animal_Tier’: [],

‘Food_Nahrung’: [],

‘Object_Gegenstand’: [],

}

### Appendix A3: subnetwork for phonological representations (python dictionary)

i) First two lines: Relation type: ‘InclPhon’: phonological representation of a word includes sub-representations (e.g., single phones, groups of phones, syllables)

ii) phonos = {}: Phonological representations of words are listed as semantic pointers in the Python dictionary phonos = {}. Each semantic pointer labels the phonological representation of a word and is listed at the beginning of a new line. Relations are listed in the same line.

Phonological representations of words include an underline in order to separate syllables. The phonological transcriptions in part use SAMPA notation (SAMPA, 2005). In addition, the prefix ‘ST_’ marks a stressed (vs. unstressed) syllable. Sub-representations (see deep phonological representations in Appendix 4) always start with the prefix ‘P_’ for part. If a sub-representation is part of a stressed syllable, the prefix ‘ST_’ is included as well. Phonological representations are always related to a word (see Appendix A1).

    inclPhon = ‘InclPhon’

Relation_types = [inclPhon]

phonos = {

‘St_El_mend’: [(inclPhon, ‘PSt_El’), (inclPhon, ‘P_mend’)],

‘St_E_pe_si’: [(inclPhon, ‘PSt_Ep’), (inclPhon, ‘PSt_E’), (inclPhon, ‘P_pe’), (inclPhon, ‘P_si’)],

‘St_E_pel’: [(inclPhon, ‘PSt_Ep’), (inclPhon, ‘PSt_E’), (inclPhon, ‘P_pel’)],

…‘St_trOm_pet’: [(inclPhon, ‘PSt_tr’), (inclPhon, ‘PSt_trOm’), (inclPhon, ‘P_pet’)],

}

### Appendix A4: subnetwork for deep phonological representations (python dictionary)

Deep phonological representations are single phones, groups of phones, syllables. They are listed as semantic pointers in the Python dictionary phonos_deep = {}. These representations always start with the prefix ‘P’ for part. If a sub-representation is part of a stressed syllable, the prefix ‘ST_’ is included as well. Deep phonological representations are related to phonological representations (Appendix A3).

    phonos_deep = {

# syllables or parts of syllables, occurring in more than one word:

‘PSt_Ep’: [], ‘PSt_bE’: [], ‘PSt_bi’: [], ‘PSt_br’: [], ‘PSt_da’: [], ‘PSt_kE’: [], ‘PSt_El’: [], ‘PSt_fl’: [], ‘PSt_lE’: [], ‘PSt_pi’: [], ‘PSt_rE’: [], ‘PSt_snE’: [],

‘PSt_sp’: [], ‘PSt_sk’: [], ‘PSt_tr’: [],

# other words or syllables, not necessarily describing dependencies between phonological representations within phonological network:

‘P_bel’: [], ‘P_bIdZ’: [], ‘P_bIt’: [], ‘P_boUn’: [], ‘P_del’: [], ‘P_der’: [], ‘P_dIN’: [], ‘P_fent’: [], ‘P_fi’: [], ‘P_fIk’: [], ‘P_je’: [], ‘P_kel’: [], ‘P_ken’: [], ‘P_kIdZ’: [], ‘P_kIt’: [], ‘P_kOt’: [], ‘P_la’: [], ‘P_le’: [], ‘P_lI’: [], ‘P_mel’: [], ‘P_mend’: [], ‘P_nat’: [], ‘P_ne’: [], ‘P_nIN’: [], ‘P_nItS’: [], ‘P_noU’: [],

‘P_pe’: [], ‘P_pel’: [], ‘P_pet’: [], ‘P_pi’: [], ‘P_pri’: [], ‘P_rel’: [], ‘P_ret’: [], ‘P_rI’: [], ‘P_si’: [], ‘P_te’: [], ‘P_tel’: [], ‘P_ten’: [], ‘P_tje’: [], ‘P_we’: [], ‘P_wen’: [], ‘P_wer’: [], ‘PSt_bas’: [], ‘PSt_bEg’: [], ‘PSt_bEn’: [], ‘PSt_bras’: [], ‘PSt_brEd’: [], ‘PSt_brEn’: [], ‘PSt_brIk’: [], ‘PSt_bU’: [], ‘PSt_bUs’: [],

‘PSt_dab’: [], ‘PSt_dak’: [], ‘PSt_daw’: [], ‘PSt_doU’: [], ‘PSt_drEs’: [], ‘PSt_dZip’: [], ‘PSt_E’: [], ‘PSt_EI’: [], ‘PSt_Elk’: [], ‘PSt_Elm’: [], ‘PSt_faI’: [],

‘PSt_flaI’: [], ‘PSt_flEg’: [], ‘PSt_fli’: [], ‘PSt_flu’: [], ‘PSt_fOks’: [], ‘PSt_hOrn’: [], ‘PSt_i’: [], ‘PSt_il’: [], ‘PSt_kaf’: [], ‘PSt_kast’: [], ‘PSt_kaUtS’: [],

‘PSt_kEI’: [], ‘PSt_kEn’: [], ‘PSt_kEs’: [], ‘PSt_kO’: [], ‘PSt_kOr’: [], ‘PSt_krIb’: [], ‘PSt_lEI’: [], ‘PSt_lEmp’: [], ‘PSt_lEn’: [], ‘PSt_mOs’: [], ‘PSt_moUl’: [],

‘PSt_muz’: [], ‘PSt_nEIl’: [], ‘PSt_pE’: [], ‘PSt_pI’: [], ‘PSt_pIg’: [], ‘PSt_pitS’: [], ‘PSt_raft’: [], ‘PSt_rEI’: [], ‘PSt_rEIk’: [], ‘PSt_rEt’: [], ‘PSt_s9rst’: [],

‘PSt_sE’: [], ‘PSt_skEIt’: [], ‘PSt_skUI’: [], ‘PSt_skUnk’: [], ‘PSt_snEIk’: [], ‘PSt_snEIl’: [], ‘PSt_snEk’: [], ‘PSt_spaI’: [], ‘PSt_spar’: [], ‘PSt_spE’: [],

‘PSt_spI’: [], ‘PSt_spun’: [], ‘PSt_straIp’: [], ‘PSt_trE’: [], ‘PSt_trEIn’: [], ‘PSt_trEk’: [], ‘PSt_trEp’: [], ‘PSt_trO’: [], ‘PSt_trOm’: [], ‘PSt_troU’: [],

‘PSt_trUk’: [], ‘PSt_tSEIn’: []

}

### Appendix A5: subnetwork for word representations (python dictionary)

Word (i.e., orthographic representations) are listed as semantic pointers in the Python dictionary words = {}. These representations always start with the prefix ‘W_’ followed by the English word.

    words = {

‘W_Almond’: [], ‘W_Apathy’: [], ‘W_Apple’: [], ‘W_Apricot’: [], ‘W_Bag’: [], ‘W_Ban’: [], ‘W_Basket’: [], ‘W_Beacon’: [], ‘W_Beaver’: [], ‘W_Bee’: [], ‘W_Beetle’: [], ‘W_Bran’: [], ‘W_Brass’: [], ‘W_Bread’: [], ‘W_Brick’: [], ‘W_Bucket’: [], ‘W_Bus’: [], ‘W_Cabbage’: [], ‘W_Cable’: [], ‘W_Calf’: [], ‘W_Camel’: [], ‘W_Candle’: [], ‘W_Carrot’: [], ‘W_Cash’: [], ‘W_Cast’: [], ‘W_Celery’: [], ‘W_Chain’: [], ‘W_Coffee’: [], ‘W_Corner’: [], ‘W_Couch’: [],

‘W_Crib’: [], ‘W_Donut’: [], ‘W_Dove’: [], ‘W_Dress’: [], ‘W_Dub’: [], ‘W_Duck’: [], ‘W_Eel’: [], ‘W_Elephant’: [], ‘W_Elk’: [], ‘W_Elm’: [], ‘W_Evening’: [], ‘W_Fire’: [], ‘W_Flag’: [], ‘W_Flea’: [], ‘W_Flu’: [], ‘W_Fly’: [], ‘W_Fox’: [], ‘W_Horn’: [], ‘W_Jeep’: [], ‘W_Ladle’: [], ‘W_Lamp’: [], ‘W_Landing’: [], ‘W_Lantern’: [], ‘W_Mole’: [], ‘W_Moose’: [], ‘W_Moth’: [], ‘W_Nail’: [], ‘W_Package’: [], ‘W_Peach’: [], ‘W_Peanut’: [], ‘W_Pecan’: [], ‘W_Piano’: [], ‘W_Pig’: [], ‘W_Rabbit’: [], ‘W_Raft’: [], ‘W_Rake’: [], ‘W_Rat’: [], ‘W_Raven’: [], ‘W_Skate’: [], ‘W_Skunk’: [], ‘W_Snack’: [], ‘W_Snail’: [], ‘W_Snake’: [], ‘W_Sparkle’: [], ‘W_Spatula’: [], ‘W_Spider’: [], ‘W_Spinach’: [], ‘W_Spoon’: [], ‘W_Squirrel’: [], ‘W_Stripe’: [], ‘W_Thirst’: [], ‘W_Tractor’: [], ‘W_Traffic’: [], ‘W_Train’: [], ‘W_Trap’: [], ‘W_Trolley’: [], ‘W_Trombone’: [], ‘W_Trophy’: [], ‘W_Truck’: [], ‘W_Trumpet’: []

}
